# Intracellular Transport of Monomeric Peptides, (Poly)Peptide-Based Coacervates and Fibrils: Mechanisms and Prospects for Drug Delivery

**DOI:** 10.3390/ijms262211015

**Published:** 2025-11-14

**Authors:** Tatiana Vedekhina, Iuliia Pavlova, Julia Svetlova, Julia Khomyakova, Anna Varizhuk

**Affiliations:** 1 Lopukhin Federal Research and Clinical Center of Physical-Chemical Medicine of Federal Medical Biological Agency, Malaya Pirogovskaya, 1a, 119435 Moscow, Russia; 2 Lomonosov Institute of Fine Chemical Technologies, MIREA–Russian Technological University, Vernadsky Avenue, 86, 119454 Moscow, Russia

**Keywords:** cell penetrating peptides, peptide-based coacervates, fibrils, direct intracellular transport, endocytosis, macropinocytosis, phagocytosis

## Abstract

Peptides are emerging as versatile platforms in medicine, serving as therapeutic agents, diagnostic probes, and drug delivery vehicles. Their physical state—in a form of monomeric cell-penetrating peptides (CPPs), liquid-like coacervates, or solid amyloid fibrils—critically determines their interaction with cell surfaces and subsequent intracellular trafficking pathways. While the transport of CPPs has been extensively studied, the mechanisms governing the cellular uptake of peptide-based coacervates and fibrils are less understood. This review summarizes the current understanding of the intracellular transport mechanisms of all three distinct peptide states and their complexes or conjugates with cargo molecules. We examine a range of pathways, including direct membrane translocation, several endocytosis subtypes, and phagocytosis-like transport. Particular attention is given to unique aspects observed exclusively for CPPs, coacervates, or fibrils. Further verification and detailed characterization of internalization mechanisms are crucial for the rational design of next-generation peptide-based carriers that allow for precise cargo delivery and therapeutic efficacy.

## 1. Introduction

Peptides are increasingly being used as therapeutic agents. They can act as competitors or mimics of endogenous metabolites, including hormones, neurotransmitters, ion channel ligands, etc. [1]. In diagnostics, peptides are integral to various immunoassay platforms [2] and often serve as antibody substitutes [3]. Significant advances have also recently been made in the development of peptide-based drug carriers and vaccines [4]. Therapeutically relevant peptides can be classified in multiple ways, e.g., based on their function, origin, or design/selection strategy. Most widely used peptides are obtained from natural products or selected using phage display and other screening methods [5,6]. However, structure-guided rational design and computational approaches [7,8,9], including those aided by artificial intelligence [10], are currently yielding multiple new, promising alternatives.

This review focuses on the peptide-cargo intracellular entry pathways, which are determined by the size and physicochemical properties of the internalized molecules. To overcome the limited cargo-loading potential of small monomeric CPPs, multimeric peptide associates and aggregates capable of encapsulating multiple cargo molecules have been proposed. The increased size and distinct physicochemical properties (Table 1) of these peptide associates and aggregates suggest different membranotropism, entry pathways, and intracellular distribution. Therefore, peptide oligomerization state and phase transitions should be considered when developing a peptide-based therapeutic or selecting a cargo-loading platform. Therapeutically relevant peptides typically exist in one of the following states:

- Monomeric/oligomeric cell-penetrating peptides (CPPs);
- Multimeric dense but liquid peptide associates, termed coacervates;
- Multimeric solid aggregates, including cross-beta structures (fibrils).

**Figure 1:**
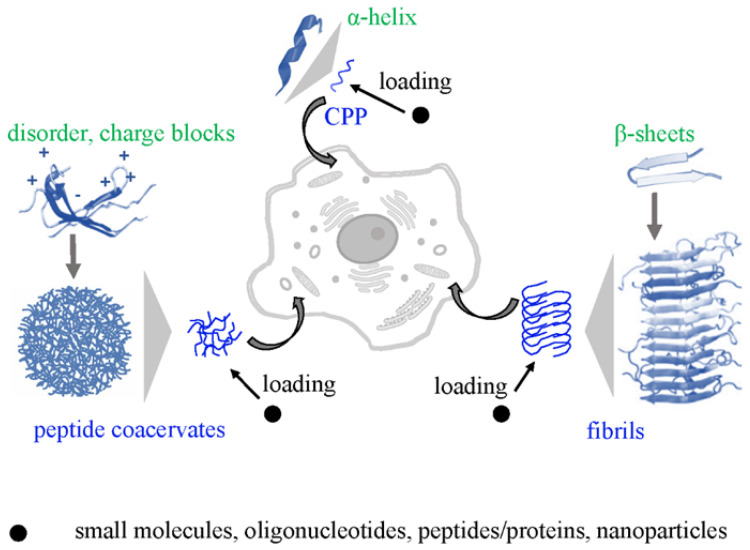
Peptide-based vehicles: CPPs, coacervates and fibrils (blue), their characteristic secondary structures features (green), and cargo (black).

Developing drug carriers based on CPPs, coacervates, and fibrils requires full control of their intracellular transport. Thus far, only the underlying mechanisms of CPP internalization have been reviewed [11]. Here, we compare CPPs with coacervates and fibrils (Figure 1) and discuss the key steps of intracellular transport in relation to different peptide phase states and their applicability to cargo delivery.

## 2. Cell Penetrating Peptides (CPPs), Coacervates and Fibrils

CPPs are short (4–40 aa) native or synthetic peptides that enter cells in the absence of vehicles and facilitate the uptake of peptide-associated molecules/nanoparticles. Native CPPs have been derived from various organisms, ranging from viruses to vertebrates. These peptides can be produced in vivo through the partial proteolysis of parent proteins, and they are able to perform regulatory functions in their native contexts. Examples of native CPPs derived from viruses include fragments of the herpes simplex virus tegument protein (VP22 peptide [12]), HIV transactivator of transcription (TAT peptide [13,14]), HIV glycoprotein gp41 (gp41 peptide [15]), influenza virus hemagglutinin (KALA peptide [16] which was updated to Hel 11-7 peptide [17]), etc. Examples of insect- and vertebrate-derived CPPs include fragments of the transcription factor Antennapedia of *Drosophila melanogaster* (penetratin, also known as pANTP peptide [18]), human Kaposi’s sarcoma-associated glycosylated fibroblast growth factor (FGF peptide [19]); caiman immunoglobulin light chain (Ig(v) peptide [20]), etc. Sequence analysis has revealed no significant homology among CPPs. However, a consistent feature that has been observed is the presence of multiple arginine residues. Most of the CPPs are cationic (e.g., TAT and oligo-R), hydrophobic (FGF, gp41 and Ig(v) peptides), or amphipathic (Hel 11-7, VP22, pANTP, transportan and its derivative TP10 [21]). Classic examples of amphipathic peptides are MPG and Pep-1, obtained by fusing a hydrophobic motif and a K-rich nuclear localization signal through a short linker [22]. Rational design of artificial amphipathic peptides typically suggests alternation of hydrophobic and cationic residues. A notable example is the family of P-rich peptides (XZZ)_3_, where X is a hydrophobic amino acid residue, and Z is the guanidinium-proline residue [23]. Alternatively, amphipathic CPPs can be obtained by conjugating hydrophobic CPPs with charged residues or membranotropic amphipathic ones, such as fatty acids. A representative example is a stearyl-TP10 derivative PF14 [24]. There are a few notable examples of anionic CPPs, including the plant-derived peptide rT7 [25] and the artificial peptide p28 [26], which preferentially enters cancer cells. Most CPPs are too short to fold into structural motifs. However, they may contain alpha-helical elements or beta-sheets. The former are particularly prevalent [27].

CPPs serve as potent molecular shuttles that enable the intracellular delivery of a wide range of biologically active cargo, such as small molecules, polypeptides, oligonucleotides, and nanoparticles [28,29]. Unlike electroporation or microinjection, CPP-mediated delivery can be performed in vivo and enables cargo distribution across diverse tissues [30,31]. CPPs exhibit low toxicity and show promise for enhancing the effectiveness of anti-inflammatory, anticancer, antimicrobial, neuroprotective, and antiviral agents [32,33,34,35]. For example, insulin delivery using CPPs, such as TAT, pVEC, penetratin, and octolycin, enhances drug bioavailability during intestinal absorption [36]. Another notable CPP application is the development of CPP-antigen fusion proteins for human or animal vaccination [37]. CPPs have also successfully delivered genome editing tools into hard-to-transfect cells, like Jurkat and NK cells [38,39]. For instance, the His-tagged fusion of the endosomolytic fragment CM18 and a TAT derivative PTD4 delivered CRISPR/Cas9 and CRISPR/Cpf1 systems more efficiently than the lipid-based reagent CRISPRMax lipofectamine [38].

Coacervates are moderately dense droplet-like associates of peptides or other biopolymers that form through liquid–liquid phase separation (LLPS) [40,41,42]. Above the critical peptide concentration, a previously uniform system demixes into two phases: a condensed phase enriched with peptides and other components, and a bulk solution. An important property of peptide coacervates is their tunable composition and the ability to encapsulate various molecules, ranging from large proteins and oligonucleotides to low-molecular weight therapeutics and dyes [43]. Peptides that undergo coacervation are usually longer than monomeric CPPs and often contain low-complexity regions that promote conformational disorder and repeated sequences with charge blockiness that facilitate transient electrostatic interactions [44]. The molecular grammar of coacervates is similar to that of their endogenous analogs, biocondensates. Coacervation-prone peptides resemble the intrinsically disordered regions of condensate scaffolding proteins [45]. In addition to electrostatic interactions, the common types of contacts in both coacervates and peptides include cation-pi and pi-pi interactions [46,47] (e.g., those between R/K and/or aromatic amino acid residues). The compositional similarity raises concerns about possible interference of coacervates with biocondensate dynamics. For instance, coacervates that serve as nucleic acid carriers could theoretically compete with condensates involved in nucleic acids packaging or processing. However, except for stress granules, key condensates of this type are localized in the nucleus [48], whereas coacervates are typically designed to disassemble in the cytoplasm [49,50]. Furthermore, the partitioning of nucleic acids into condensates is usually a combined effect of sequence-independent transient contacts with disordered protein regions and sequence recognition by structured domains of condensate-scaffolding proteins [51]. Unlike endogenous proteins, coacervation-prone peptides lack structured domains.

Coacervates have recently garnered significant attention as delivery systems for biomacromolecules [50,51,52,53]. For instance, coacervates of R-rich peptides, such as tandem pentapeptide repeats (RRASL)_1–3_ [54], are particularly adept at encapsulating nucleic acids and can be assembled via pH adjustment [55,56,57]. In contrast, temperature-sensitive coacervates formed by mostly uncharged elastin-like G-rich peptides (e.g., tandem repeats (VPGXG)_n_, where X is any amino acid except proline) are typically assembled through cooling/heating cycles and do not readily incorporate charged cargo. However, the elastin-like peptides retain the propensity for coacervation when conjugated with therapeutic oligonucleotides and can be used in delivery systems [58]. Coacervates of peptides with tandem GHGXY repeats (HB*pep*, X = L/P/V) can incorporate a broad range of macromolecular therapeutics, including nucleic acids and hormones such as insulin [52]. The presence of histidine residues enables their extracellular assembly in the convenient (near-physiological) pH range. Incorporating other charged, aromatic or modified amino acid residues allows for fine-tuning of cargo loading and intracellular disassembly. For instance, the HB*pep* variant with modified residues that form S-S bridges has been shown to assemble and disassemble in a redox-dependent manner [59]. Another HB*pep* variant, GW26, the 26-mer (GHGXY)_5_W, has demonstrated potential applicability for photothermal therapy [50]. The coacervates of this peptide, loaded with an anticancer agent, a photosensitizer (chlorin e6), and gold nanoparticles were delivered to cancer cells and released the cargo upon the disassembly due to the laser-induced local surface plasmon resonance. The coacervates are supposedly non-immunogenic. This has been verified for HB*pep* variants (GHGLY)_4_ and (GHGVY)_4_, using marrow-derived dendritic cells [60]. The coacervates did not affect the production of cytokines, including pro-inflammatory (IL-6, TNF-α), tolerance-inducing (IL-10), and maturation-specific (IL-15) ones. They also did not affect the production of immunomodulating growth factors (GM-CSF, EGF, MIF, and PDGF-BB).

Amyloid fibrils are solid, highly ordered, and remarkably stable assemblies [61,62] of peptides, such as amyloid beta peptides (Aβ) [63], or proteins, such as alpha-synuclein (α-Syn) [64], tau [65,66], huntingtin [67], lysozyme [68], or prion protein PrP^Sc^ [69]. Their characteristic structural feature is the cross-beta core, i.e., arrays of H-bonded β-sheets twisting around a central axis [70]. Unlike the formation of amorphous aggregates, which is kinetically favorable, fibril assembly is thermodynamically favorable; its rate-limiting step is nucleation [71]. This step yields oligomeric protofilaments, which rapidly elongate into multimeric ones and can eventually form filaments [72]. A large group of amyloidization-prone peptides includes repetitive sequences found in natural hydrogels from sea organisms [73]. These peptides and their derivatives are suitable for preparing fibril-based 3D nanoscaffolds. They follow the (ABAC)_n_ or (ABAB)_n_ sequence rules, where A and B indicate oppositely charged or charged and aromatic amino acid residues, respectively.

Amyloid fibrils have long been regarded as pathology-specific toxic structures and products of aberrant folding resulting from dysregulated protein processing or mutations. However, the ability to form amyloid-like structures is likely a fundamental property of many native nonpathogenic polypeptides [74,75] containing amyloidogenic patterns (motifs of self-associating beta-strands) [74]. Relatively short sequences with a confirmed propensity for amyloidization can be regarded as amyloidogenic tags. They can be added to peptide/protein sequences of interest to facilitate their packaging into an amyloid-based depo for developing long-acting formulations [76,77,78]. Key prerequisites for this approach include ensuring that the released monomers remain functionally active and that the target module does not interfere with the assembly of the amyloid core. This approach has been successfully applied for delivering the gonadotropin-releasing hormone [79]. The clinical success of the resulting long-acting hormone analogs like degarelix and ganirelix demonstrates the therapeutic viability of the amyloid tag-based approach [76]. A related approach, which involves composite drug delivery platforms, namely those formed by integrating liposomes into an amyloid hydrogel network, has been shown to enhance the efficacy of hydrophilic drug payloads [80].

In addition to the tag- and composite-based approaches, traditional approaches based on to cargo loading through post-synthetic conjugation or non-covalent interactions with fibril functional groups are being developed. (Poly)peptide-based fibrils and other nanoscaffolds are being considered for delivering therapeutic proteins and have been successfully used for delivering anticancer small molecules [81]. Similarly, fibrillation-prone proteins are being considered for delivering small molecule drugs. For example, lysozyme fibrils have recently been tested as vehicles for anticancer nucleoside mimetics (antimetabolites) [82]. Due to the documented cytotoxicity of fibrils and other protein aggregates toward cancerous cells, concomitant cytotoxicity assays are integral to these studies [83]. Unlike most CPPs and coacervates, fibrils can be immunogenic, suggesting their usability as adjuvants [84,85]. Moreover, they can serve dual purposes–e.g., as act both adjuvants and antigen carriers [86]. Increasing the fibrillation potential of antigenic peptides (e.g., through point mutations that stabilize the potential beta-sheets) can dramatically improve the efficacy of peptide vaccines. A proof-of concept study has recently been reported for the G33 peptide derived from the Ebola virus glycoprotein, which is involved in the host binding and entry [87]. This peptide was rendered fibrillation-prone by a single terminal mutation and elicited a much stronger immune response than the native counterpart. Importantly, Ebola virions enter host cells via endocytosis driven by the glycoprotein-receptor interactions, whereas the isolated glycoprotein and its derivatives, including fibrils, likely utilize receptor-independent pathways [88], which need to be elucidated for future vaccine development.

To summarize this section, therapeutically relevant (poly)peptides that exist in different physical states show different characteristic features, such as charge distribution and common secondary structure elements. The key features are outlined in Table 1. The physical state determines the size range, which in turn affects the likelihood of various entry pathways, immunogenicity, and toxicity. Criteria for immunogenicity of monomeric peptides have been reviewed elsewhere [89,90,91] and are not met by most CPPs; the occasional exceptions may be attributed to impurities [92]. Similarly, although CPPs per se are generally non-toxic, the seeming exceptions may result from conjugation with cargo or other additional modules [93,94,95]. The switch from non-immunogenic monomers to immunogenic fibrils [96] is partly due to an increase in size: larger particles are more likely to be phagocytosed/macropinocytosed by antigen-presenting cells. The most intriguing open question is arguably the difference in immunogenicity between coacervates and fibrils, which have different shapes but comparable linear sizes. Hopefully, some insight into this matter will be obtained from the studies of native peptides and proteins that undergo a coacervate-to-fibril transition, such as fragments of heterogenous nuclear ribonucleoproteins or α-syn [97,98,99].

## 3. Direct Intracellular Transport of CPPs and Fibril Transport Through Tunneling Nanotubes (TNTs)

**Figure 2:**
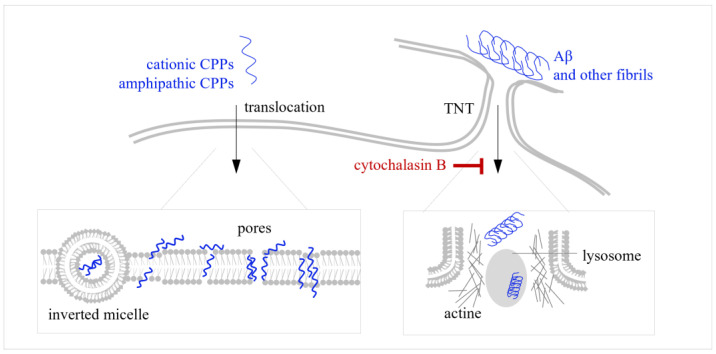
Direct intracellular transport of CPPs and fibril transport through tunneling nanotubes (TNTs). (**Left**): Schematic representation of CPP transport via membrane translocation. Cationic CPPs, such as oligo-R or TAT and its derivative PTD4 form electrostatic contacts with the negatively charged membrane surface, which results in membrane destabilization (pore formation) and direct CPP internalization. Amphiphatic CPPs, such as penetratin or transportan, initially contact the membrane surface through cationic amino acid residues. Their accumulation enables contacts between aromatic amino acid residues and lipid tails, which provokes membrane bending and the formation of inverted micelles, whose intracellular parts can be eventually disrupted to release the peptide into the cytosol. (**Right**): Schematic representation of TNT-dependent direct intercellular transport of (poly)peptide fibrils. TNTs, membrane protrusions observed in neural cells, cancer cells, monocytes, and several other cell types, enable intercellular exchange of signaling molecules and pathogens (fibrils), as well as organelles (lysosomes and mitochondria), which can also be loaded with fibrils, in a filament-dependent of independent manner. Because TNT formation is actin-dependent, the TNT-dependent transport can be inhibited by actin depolarizing drugs, such as cytochalasin B.

Direct transport (Figure 2, left) is usually regarded as a one-step process because long-lived intermediate states are absent. It can be initiated by electrostatic attraction between the CPPs and the negatively charged phospholipid membrane surface [100]. This may lead to the formation of transient pores [101] or the reorganization of the lipid bilayer into noncanonical structures, such as inverted micelles [102].

First proposed in 1985 [103], the inverted micelle model offers a mechanism for the direct translocation of amphipathic CPPs like pANTP [33]. The model posits that the CPPs initially accumulate on the plasma membrane via the electrostatic attraction of their basic residues to anionic phospholipids. Subsequently, hydrophobic amino acid residues induce local lipid reorganization. This alters the curvature of the membrane, resulting in the formation of a spherical inverted micelle structure [102,104,105]. The peptide is then released into the cytosol when the micelle disintegrates on the inner membrane leaflet. A key distinction of this model is that the peptide remains at the membrane interface without embedding in the hydrophobic core. This contrasts with pore formation models that suggest direct peptide insertion into the bilayer [106].

Transient membrane pores are commonly induced by cationic peptides, such as TAT, oligo-R peptides [107] or their analogs with shortened side chains, such as mR8 [108]. This process is driven by electrostatic interactions, and the translocation efficiency of cationic peptides correlates strongly with their net positive charge and the negative charge density of the membrane, rather than with the specific architecture of the peptide backbone [109]. At local CPP concentrations above the threshold value, the positively charged CPP side chains at the outer leaflet attract the phosphate groups of the inner bilayer leaflet. This leads to the thinning of the lipid bilayer, the penetration of water molecules through it, the enhanced lipid diffusion between the leaflets, and the formation of a transient pore through which CPPs diffuse [107]. Unlike cationic CPPs, amphipathic CPPs not only induce pore formation, but also stabilize the pores. A well-known example is the amphipathic CPP TP10 [110]. It targets membrane regions with the least dense lipid packing and folds into an α-helix upon association with the lipids. This process reinforces the bilayer defects. Additionally, electrostatic interactions with CPPs promote lateral membrane reorganization, exacerbate thinning, and eventually lead to the formation of a CPP-lined pore [111]. The self-association of CPPs on the pore surface reduces local surface tension, allowing for additional CPPs to penetrate the membrane through stabilized pores [112,113].

Direct transport through pores and inverted micelles is only possible for monomeric CPPs. Although CPPs can undergo oligomerization and even aggregation upon accumulation at the membrane surface, preformed aggregates, including fibrils, do not induce formation of pores or micelles. However, fibrils can enter cells through a distinct direct pathway: cell-to-cell transmission via tunneling nanotubes (TNTs) (Figure 2, right). The formation of TNTs is associated with the intercellular communication and underlies the spread of pathogenic fibrils of Aβ peptides, α-Syn, tau, etc. [114,115,116]. This suggests that TNTs may promote the distribution of fibril-based therapeutic composites. TNTs are thin, actin-rich membranous conduits that allow for the transport of both molecules and organelles, such as endosomes, lysosomes, and mitochondria [117]. Fibrils can pass TNTs in a free state, within lysosomes [118,119], or within mitochondria [120]. For most pathogenic and therapeutic fibrils, the contribution of this mechanism to fibril propagation awaits further investigation. For its verification, semi-specific TNT inhibitors, such as cytochalasin B, can be considered. Cytochalasin B prevents TNT formation without affecting endocytosis and phagocytosis [121] and has been used to confirm TNT-dependence of the propagation of α-Syn protofibrils [122].

Several factors can explain the slow progress in studying fibril transport through TNTs. First, TNTs are polymorphic [123] and show varying sensitivity to microtubule modulators and, likely, to actin-depolarizing toxins such, as cytochalasin B. Second, discriminating TNTs from other membrane protrusions such as [124] requires high resolution imaging and advanced fluorescence-based approaches [125,126]. In contrast, transient membrane pores can be verified using simple fluorescence techniques [127], and the contribution of pore formation to CPP transport is well-established.

## 4. Clathrin- and Dynamin-Mediated Endocytosis of CPPs and Fibrils

**Figure 3:**
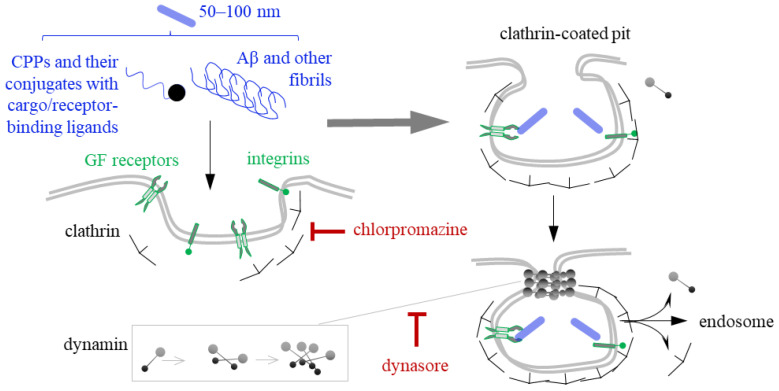
Clathrin- and dynamin-mediated endocytosis (CME) of CPPs and fibrils. CME is initiated by interactions between conjugates or fibrils and growth factor (GF) receptors, scavenger receptors, or other specific receptors. These interactions promote membrane invagination and the assembly of a clathrin lattice around the pit/vesicle. This process can be blocked by chlorpromazine. Separating the lattice-coated vesicle from the cytoplasmic membrane requires GTP-dependent polymerization of dynamin, which can be blocked by dynasore. Subsequent uncoating of the vesicle yields early endosomes, which mature into late endosomes and, eventually, lysosomes. The cargo may end up in the lysosomes unless the conjugate contains endosomal escape-promoting motifs.

Clathrin-mediated endocytosis (CME) is a primary, highly selective route for internalizing macromolecules and complexes up to 100 nm in size [128]. Most CPP-cargo complexes and some fibrils fall within this size range and are susceptible to CME. This process is orchestrated by actin, clathrin, and dynamin (Figure 3). Actin supports membrane invagination, which initiates pit formation. Clathrin is recruited to the pit in response to receptor activation, and its oligomerization facilitates maturation of the pit into a clathrin-coated vehicle. Dynamin then facilitates scission of the clathrin-coated vesicle from the plasma membrane [129]. This is typically followed by the disassembly of the clathrin lattice [130]. The internalized peptide-receptor complexes are then directed into intraluminal vesicles within multivesicular bodies (late endosomes) and are eventually degraded upon fusion with lysosomes.

CME is typically verified using specific inhibitors of clathrin assembly and dynamin oligomerization [131], such as chlorpromazine [132] and dynasore [133]. Examples of CPPs whose conjugates with cargo exhibit a propensity for CME include amphipathic fragments of the tumor suppressor p14ARF [32], TAT derivatives [134], anionic CPPs [135], and some others [136]. This propensity may be determined by affinity for receptors associated with clathrin recruitment, such as growth factor receptors, scavenger receptors, and others [137]. Considerable research is dedicated to engineering targeted delivery platforms that utilize CPPs with inherent selectivity for CME-linked receptors that are prevalent on the surface of cancer cells [138,139].

CME may be the primary route of internalization for Aβ, α-Syn, tau, and huntingtin fibrils [119,140,141,142,143]. The most conclusive evidence has been obtained for α-Syn. First, its internalized fibrils colocalize with key early endosome markers such as EEA1 and Rab5 [119,144], as well as with late endosome and lysosome markers such as Rab7 and LAMP1 [144,145]. Second, reducing the incubation temperature diminishes fibril uptake, which is consistent with the temperature dependence of CME [146]. Third, genetic impairment of dynamin function or chemical inhibition using dynasore reduces internalization [119].

Fibril uptake supposedly depends on scavenger receptors, such as low-density lipoprotein receptor-related protein 1 (LRP1). LRP1 is expressed in numerous cell types beyond neurons, including epithelial and muscle cells, fibroblasts, retinal Müller glial cells, monocytes, macrophages, hepatocytes, adipocytes, vascular smooth muscle cells, and cancer cells. It is typically present in raft-free membrane regions [147] and can be activated by coreceptors, specifically heparan sulfate proteoglycans (HSPGs) [148], which are expressed throughout the body [119]. Binding to HSPGs and subsequent internalization has been reported for Aβ, tau, and α-Syn fibrils [149], and α-Syn fibrils [150]. Following internalization, fibrils accumulate within lysosomes. The precise mechanism by which they escape lysosomes remains unclear. One possible explanation is that the fibrils induce lysosome rupture [141]. The accumulation of endogenous fibrils, which can propagate in a prion-like manner during the development of neurodegenerative diseases, induces lysosomal stress [151]. This exacerbates fibril toxicity and causes symptoms similar to those of the lysosome storage diseases [152]. In contrast, cargo-loaded synthetic fibrils lack endogenous partners and are not expected to propagate. When introduced at moderate concentrations, they are unlikely to induce stress, and their ability to partially disrupt lysosome membranes seems beneficial, as it facilitates cargo release [141]. This is an important advantage of fibrils over the most of the CPPs in terms of delivering macromolecules within the CME-compatible size range.

Most CPPs fail to escape endosomes or lysosomes [153], so their conjugates require additional escape modules [154], e.g., hydrophobic [155] or anionic ones [156]. Notable exceptions are cyclic peptides [157]. They induce budding of the endosome membrane, forming lipid-coated vehicles that degrade shortly after to releasing the peptide and the cargo.

## 5. Caveolin- and Raft-Mediated Endocytosis of CPPs, Coacervates and Fibrils

**Figure 4:**
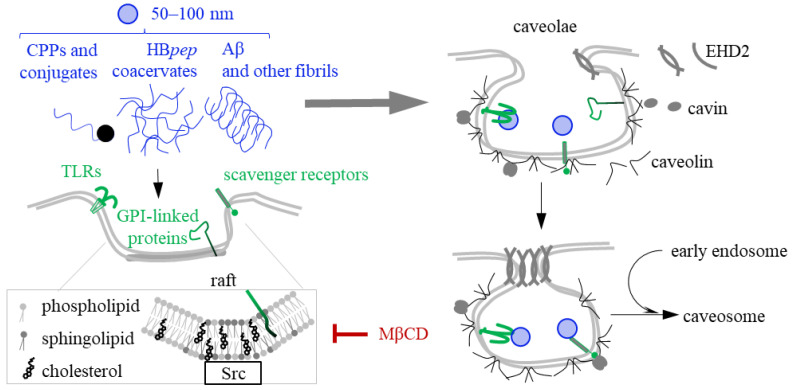
Caveolin- and raft-mediated endocytosis of CPPs, coacervates and fibrils. CvME begins with interactions between CPP-cargo conjugates, coacervates, or fibrils and GPI-anchored proteins, toll-like receptors (TLRs), scavenger receptors, or other pattern recognition receptors. These interactions cause receptor clustering and the activation of Src or other kinases, which then phosphorylate caveolin, promoting its self-assembly on the surface of the invaginated membrane (caveolae). Receptor clustering and caveolae formation depend on the local membrane lipid content. They require the presence of lipid rafts enriched with sphingolipids and cholesterol, and they can be inhibited by cholesterol-depleting agents such as methyl-β-cyclodextrin (MβCD). Caveolin, the lipid-bound inner component of the caveolae coating, interacts with cavins, which constitute the outer coating layer. The separation of the caveolar membrane from the cytoplasmic membrane may be dynamin-dependent. However, in most cases, this process requires a distinct protein, EHD2, which clusters at the neck of the coated caveolae to promote vehicle scissoring. Unlike clathrin-coated vesicles, caveolin-cavin-coated vesicles do not rapidly disassemble, and they may merge with endosomes to form caveosomes.

Caveolin-mediated endocytosis (CvME) is characterized by the formation of flask-shaped invaginations of the plasma membrane called caveolae. They form within lipid rafts, which are dynamic plasma membrane domains measuring up to 200 nm in diameter and enriched with sphingolipids, cholesterol, and ceramides (Figure 4). Caveolae formation requires structural and regulatory proteins, including caveolins [158], cavins [159], actin [160] and others [161]. CvME begins with the receptor-dependent activation of membrane-associated kinases, such as Src [162,163], which enables caveolin recruitment and self-association. The subsequent steps are generally analogous to those of CME. They include the separation of coated caveola from the membrane and its possible fusion with early endosomes. However, unlike clathrin-coated vesicles, caveolae and their cargo often bypass lysosomes [164], enabling the cargo to avoid degradation.

Since the first step of CvME requires the presence of rafts, the entire process is raft-dependent and can be inhibited by cholesterol-depleting agents, such as methyl-β-cyclodextrin (MβCD) [165]. However, the term “raft-dependent endocytosis” is often used to describe a distinct mechanism, namely the caveolin-independent internalization of positively charged lipid vesicles or enveloped viruses [166] after their nonspecific adsorption to the negatively charged cellular surface [167,168]. Additionally, there are at least two types of caveolin- and raft-dependent endocytosis. One type is modulated by the recruitment of endophilin A2 and actin polymerization in response to receptor activation [169]. It also requires dynamin to separate caveolae from the plasma membrane [170]. The other utilizes glycoproteins, glycolipids, or their conjugates–e.g., proteins anchored to glycosylphosphatidylinositol (GPI)–to initiate caveolin assembly [171]. It requires EH domain-containing protein 2 (EHD2) rather than dynamin to separate caveolae from the plasma membrane.

CPPs are too small for CvME, and the CvME potential of their cargo conjugates depends on the size and nature of the cargo. Transportan and the TAT peptide only undergo CvME when conjugated to macromolecules [172,173,174] that can be recognized by GPI-anchored proteins, scavenger receptors or other CvME-associated receptors [175,176]. CvME-prone CPP-protein conjugates are gaining attention in the development of novel immunotherapeutic agents, such as cancer vaccines. To prolong antigen presentation, tumor-specific antigens are fused with CPPs to ensure efficient delivery to CD4+ and CD8+ T cells. Such vaccine candidates show promise in preclinical studies [177]. For instance, fusing a multi-epitope antigen with the Z12 CPP, which is derived from the Epstein–Barr virus protein ZEBRA, yielded a vaccine candidate that elicited a robust antitumor immune response in mice following intranasal administration [178].

The mechanisms by which coacervates cross the cellular membrane are largely understudied. The possibility of their endocytosis is a matter of active debate. Coacervates typically range in size from 300 nm to 1 μm [179], which falls within the endocytosis-compatible range of up to several μm [180], but exceeds the average size of CME- and CvME-prone molecules (50–100 nm). Early studies of coacervate uptake revealed sensitivity to the lipid content, particularly cholesterol, so the proposed mechanism was defined as raft-dependent endocytosis. However, those studies used model objects, namely liposomes or giant unilamellar vesicles [55,56], rather than actual cells. Additionally, they focused on oligo-R and spermine-based coacervates [55], while coacervates based on pentapeptide repeats were not tested. Subsequent studies of HB*pep*-based coacervates in cells supported a distinct mechanism reminiscent of micropinocytosis and phagocytosis [181]. However, raft-dependent endocytosis could not be excluded.

Experiments with liposomes [56] revealed two key factors that determine coacervate-bilayer interactions and the internalization pathway of coacervates: the ζ-potential difference between the membrane and the coacervate and the extent of lipid partitioning into the coacervate (Kp). At low Kp values (<75), electrostatic interactions dominate coacervate-liposome mixing; the coacervates “wet” the membrane, but internalization is limited. Conversely, coacervates with high Kp (>75), such as oligo-R based ones, demonstrate efficient lipid absorption and superior transmembrane penetration capability. Whether the Kp rule applies to HB*pep*-based coacervates awaits clarification. Such coacervates are expected to exhibit a partial positive charge at pH 7.5. Consequently, both adhesion through electrostatic interactions and coacervate-lipid mixing are possible. Interestingly, HB*pep*-based coacervates cross model membranes with 20 mol% cholesterol particularly efficiently [181], probably due to the optimal membrane fluidity for coacervate attachment [182,183].

Recent studies of coacervates based on and RRASL repeats [184], VPGXG repeats (elastin-like peptides) [185], and HB*pep* [181] in cell cultures revealed sensitivity of their uptake to MβCD pretreatment. These findings support CvME/raft-dependent internalization. In contrast, pretreatment with the clathrin polymerization inhibitor chlorpromazine [60,185] or a dynamin inhibitor [60] has no significant effects. The uptake was primarily analyzed by flow cytometry using labeled peptides (e.g., those conjugated with tetraphenylethylene, a fluorophore with an aggregation-induced emission). For HB*pep* coacervates, an additional verification was obtained using an in vitro antigen presentation assay [60]. Dendritic cells were treated with HB*pep*-based coacervates containing the model antigen ovalbumin under various conditions. Then, they were co-cultured with hybridoma cells, and the secretion of interleukin IL-2 was used to evaluate the efficiency of the antigen uptake. The results agree with those of the flow cytometry assays [181]: both methods confirm the role of the cholesterol content. In summary, coacervates enter cells in a raft-dependent manner, likely through a unique endocytosis-like mechanism. CME can be excluded, but CvME might be possible, though no evidence of caveolae formation has been reported so far.

In the case of fibrils, neither CME nor CvME/raft-dependent endocytosis can be ruled out. Conclusive evidence has only been obtained for raft-dependent endocytosis initiated by nonspecific electrostatic attraction to the cell surface [148]. These interactions have been characterized in particular detail for α-Syn [119], whose N-terminal domain contains positively charged residues that are critical for its association with anionic membrane components [186]. Apart from endocytosis, these interactions may facilitate direct entry in a CPP-like manner. Specifically, they induce conformational changes in the membrane [187] and may promote the formation of membrane-embedded pores and annular α-Syn structures within lipid bilayer [188], which compromises membrane integrity and accounts for fibril toxicity. Alternatively, the initial nonspecific electrostatic attachment can facilitate fibril recognition by specific receptors [119], including raft-sensitive ones like lymphocyte-activation gene 3 (LAG3) and Toll-like receptors (TLRs), as well as scavenger receptors like CD36 [144,189]. CD36 is widely expressed in immune and non-immune cells and shows affinity for Aβ fibrils and apolipoprotein C-II fibrils [190]. LAG3 is expressed in neurons, peripheral immune cells, and dendritic cells [119,191] and shows affinity for α-Syn fibril [144]. TLRs are expressed in immune and several types of non-immune cells, including microglia, neurons, astrocytes, and oligodendrocytes. TLR2 and TLR4 bind to α-Syn fibrils and facilitate their uptake by microglia [189], which may contribute to the development of pathology upon α-synuclein aggregation [119].

In summary, sensitivity to lipid rafts has been reported for peptides in all three phase states. However, it remains unclear whether their raft-dependent entry can be classified as CvME remains unknown. Direct evidence has only been obtained for CPP-cargo conjugates [172,173,174], and, in these cases, CvME prevalence was due to cargo features rather than CPP features. Analysis of these examples suggests that the CPP conjugates (but probably not fibrils/coacervates) can be programmed for CvME to facilitate lysosome by-pass by incorporating ligands to GPI-anchored proteins/scavenger receptors [176]. This approach can be regarded as an alternative to incorporating lysosome escape modules into CME-prone conjugates.

## 6. Macropinocytosis of CPPs, Coacervates and Fibrils

**Figure 5:**
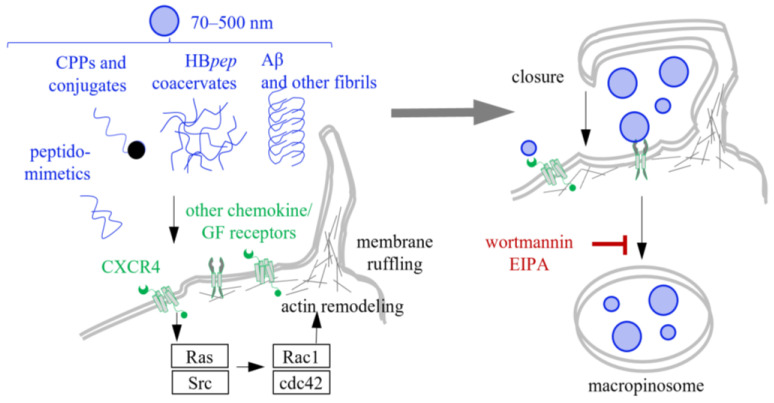
Macropinocytosis of CPPs, coacervates and fibrils. Macropinocytosis can be nonspecific or activated by the interaction of CPPs, coacervates, or fibrils with chemokine receptors, such as CXCR4, which affect membrane-associated kinases, including Ras and Src. These kinases then activate the downstream effectors of the signaling pathway that promotes actin polymerization (Rac1 and cdc42). Actin remodeling then triggers membrane ruffling and protrusion formation. The closure of these protrusions enables the engulfment of surrounding fluid containing CPPs, coacervates, or fibrils. This process can be blocked by specific inhibitors such as wortmannin or the amiloride derivative ethylisopropylamiloride (EIPA). The engulfed peptides end up in micrometer-sized macropinosomes, which can mature into lysosomes.

Macropinocytosis is a nonspecific, actin-dependent form of endocytosis that facilitates the internalization of extracellular media into large (micron-sized) vacuoles known as macropinosomes [192]. Although macropinocytosis is primarily responsible for fluid uptake, it can also facilitate the cellular entry of CPPs, coacervates, and fibrils [193] (Figure 5). Macropinocytosis typically accompanies membrane ruffling, which is the formation of dynamic membrane folds and protrusions induced by actin polymerization [194]. These ruffles can retract to restore a smooth membrane surface or fold back onto themselves, encapsulating extracellular fluid and membrane-bound molecules into macropinosomes [195,196]. This process resembles raft-dependent endocytosis. However, macropinosomes are formed from large areas of the plasma membrane that include rafts, but are not exclusively composed of them [195]. A key distinction from CME and CvME is that macropinocytosis is independent of dynamin [197]. At the same time, it is particularly dependent on the regulators of actin reorganization [193], both direct and indirect. Examples of the former include cell division control protein 42 (Cdc42) and Rho GTPase Rac 1 [198,199]. Examples of the latter include Ras and Src kinases, which affect Rac1 and Cdc42 and can be activated by peptide interactions with growth factor receptors and GPI-anchored proteins [200].

Macropinocytosis of CPPs can result from their nonspecific interactions with membrane components [201,202,203], e.g., those expected for fatty acid-containing CPP derivatives, such as PF14, in CPP-cargo complexes [24,204]. Alternatively, it can be activated via signaling cascades that promote actin filament growth and are triggered by CPP interactions with chemokine receptors (e.g., CXCR-4 [203]), HSPGs and other proteoglycans [205], etc. [206]. Examples of CPPs that reportedly enter cells via macropinocytosis include TAT [207], oligo-R [134,203,208,209,210], transportan derivatives, synthetic anionic CPPs [135], and CPP complexes with therapeutic macromolecules [211]. In most of these cases, the uptake mechanism was confirmed using specific macropinocytosis inhibitors, such as amiloride or its derivative ethylisopropylamiloride (EIPA), which affect sodium-proton exchangers and thus lower the submembranous pH [212], or wortmannin, which inhibits phosphatidylinositol 3-kinases (PI3K) [213]. The submembranous pH homeostasis, which is normally maintained by sodium-proton exchangers, is crucial for actin remodeling. PI3K activity, which generates phosphoinositide triphosphate, is necessary to activate phospholipase C, which generates diacylglycerol for Ras activation within membrane protrusions cup closure.

The first-generation inhibitor amiloride is relatively weak and may provide false-negative results, as was the case with coacervates based on elastin-like peptides [185] and HB*pep* variants [214]. Despite the minimal impact of amiloride on the cellular uptake of these coacervates, the more potent inhibitors wortmannin and EIPA significantly reduced coacervate uptake, supporting their internalization through macropinocytosis [60]. It remains unclear whether the wortmannin/EIPA-sensitive uptake of these coacervates is distinct from their MβCD-sensitive, raft-dependent endocytosis [181] or if there is a single, noncanonical, cholesterol-dependent, micropinocytosis-like mechanism. Moreover, the excessive membrane movements upon coacervate-induced ruffling may transiently increase the membrane permeability [215]. In other words, the presumed micropinocytosis-like uptake may be accompanied by direct transport, which explains its moderate sensitivity to specific inhibitors [185,214].

As concerns fibrils, their uptake via micropinocytosis is mediated by HSPGs, at least in the case of tau, α-Syn, and Aβ [142,216,217]. For tau fibrils, this has been demonstrated in primary neurons [216] and astrocytes [142]. The role of specific interactions was confirmed using competitive inhibitors of HSPG binding, such as heparin [216]. Macropinocytosis was confirmed using the first-generation inhibitor amiloride [218] and a fluid-phase pinocytosis marker, labeled dextran, which colocalized with labeled fibrils.

We conclude that micropinocytosis plays a non-decisive role in peptide internalization, regardless of the peptide state. Its dependence on HSPG suggests that it likely co-occurs with other subtypes of endocytosis [219]. Regarding the intracellular fate of peptides/cargo, micropinocytosis resembles CME rather than CvME. The pinocysed conjugates and coacervates must be protected from degradation and/or supplemented with an escape module in view of the eventual macropinosome-lysosome fusion. However, unlike CME, micropinocytosis suggests profound membrane dynamics, and an enhanced likelihood of spontaneous direct transport, so the conjugates/coacervates can end up in cytosole even without the escape modules.

## 7. Phagocytosis of Condensates and Fibrils

Phagocytosis is a process by which a subset of immune cells (phagocytes) engulfs large objects (>500 nm), typically bacteria and cell debris, after recognizing pathogenic patterns with respective receptors [220]. This process varies among key phagocytes, including macrophages, neutrophils, and dendritic cells [221]. In all cases, however, it includes three stages: (1) chemotaxis of the phagocyte, followed by binding to the target particle, (2) receptor clustering and signaling, and (3) particle internalization. This final stage involves cytoskeletal rearrangement and the formation of a phagocytic “cup” surrounded by protrusions, or pseudopods, that eventually close the cup, yielding a phagosome [222]. Phagosomes then fuse with lysosomes to form phagolysosomes, where the engulfed particles are digested. Therefore, phagocytosis of cargo-loaded (poly)peptide vehicles, similar to CME, suggests rapid cargo degradation, unless the peptides contain motifs that promote escape from the phagosome. Such motifs have been identified in bacterial and fungal pathogens [223,224].

**Figure 6:**
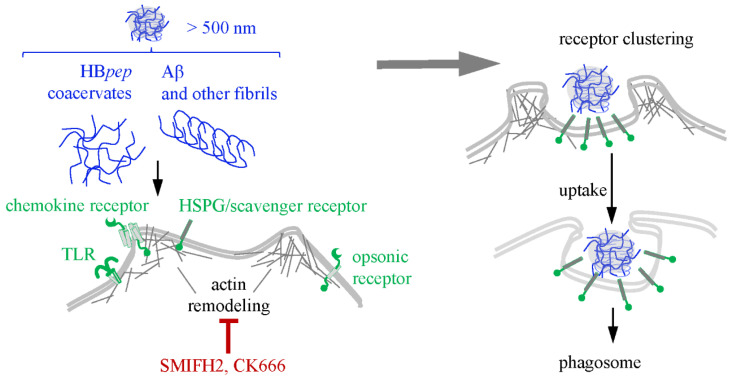
Phagocytosis of coacervates and fibrils. Phagocytosis typically requires the activation and clustering of phagocytosis-related receptors (opsonic, nonopsonic, or nonspecific ones), which triggers actin remodeling. Coacervates may trigger this process in a formin-dependent manner via interactions with lipids, while their recognition by phagocytosis-related receptors is yet to be tested. Fibrils can be recognized by nonopsonic phagocytosis-related receptors, such as HSPG and CD36, or nonspecific ones, such as TLRs. Actin remodeling yields large membrane protrusions (pseudopods) characteristic of the canonical phagocytosis or thinner ones (filopodia) characteristic of the noncanonical coacervate-induced phagocytosis. The latter process can be blocked using inhibitors of the actin related protein complex (CK666) or formin (SMIFH2). In canonical phagocytosis, phagosomes eventually fuse with lysosomes, while the outcome of the noncanonical phagocytosis awaits clarification.

Monomeric CPPs are too small to induce receptor clustering and the formation of phagocytic cups. However, both coacervates and fibrils exhibit signs of internalization via a phagocytosis-like mechanism (Figure 6). For coacervates enriched in aromatic amino acid residues, tight adhesion to the lipid bilayer followed by mechanosensitive filopodial capture may initiate this process [182,183], which suggests the redundancy of phagocyte-specific receptors. The key evidence for this noncanonical phagocytosis-like uptake was obtained through morphological characterization of HB*pep*-based coacervate engulfment by non-phagocytic HepG2 hepatocellular carcinoma cells and HeLa cervical adenocarcinoma cells [181]. The intermediates of this process were visualized using high-angle annular dark-field scanning transmission electron microscopy and scanning electron microscopy. Representative morphologies showed that the coacervates adhered to the cell surface and “sank” into filopodia-surrounded “cups.” Unlike pseudopods, which contain branched actin, filopodia critically depend on unbranched actin filaments [225], whose assembly is triggered by formin family mechanosensors [226] along with the actin related protein complex Arp2/3. Consistently, the engulfment of HB*pep*-based coacervates was sensitive [181] to the formin inhibitor SMIFH2 [227,228] and the Arp2/3 inhibitor CK666 [229]. The intracellular fate of the coacervates has not been characterized in sufficient detail. However, the cargo appears to be released freely when the coacervates disassemble efficiently under intracellular conditions, as in the case of the glutathione-sensitive HB*pep* derivatives [50].

Fibrils appear to enter immune cells via canonical phagocytosis and also modulate their phagocytic activity [230]. For example, Aβ fibrils activate the phagocytosis of microglia through interaction with scavenger receptors (CD36), integrins (β1), and other receptors [231,232]. Interestingly, this activation can be reduced by oligomeric β-amyloids. Conversely, α-Syn fibrils inhibit phagocytosis in glia and macrophages; however, these effects can be mitigated by monomeric α-Syn [230,233,234,235]. Apart from Aβ and α-Syn, phagocytosis has been observed for immunoglobulin light chain–based fibrils [236] and cannot be excluded for other cross-beta structures.

Similarly to coacervate studies, fibril phagocytosis research relies mainly on optical imaging [236]. The intracellular fate of the phagocytosed fibrils awaits clarification. How-ever, one could reasonably assume an outcome similar to that of CME, namely, the eventual escape from phagolysosomes through membrane rupture [141].

## 8. Open Questions and Limitations the Internalization Studies

As outlined in the previous sections, cell-penetrating peptides, coacervates, and fibrils appear to exploit multiple pathways for intracellular entry. In some cases, the prevalence of a particular mechanism can be explained by the affinity of the peptides or the cargo for specific receptors. In most cases, internalization through various pathways likely occurs simultaneously. Representative examples are provided in Table 2. It should be noted that the identification of the likely pathway in these examples relied mainly on the effects of small-molecule inhibitors of supposedly specific pathway steps (Table 2). Such analyses have several notable limitations.

Ideally, the full set of pathways and orthogonal inhibitors should be considered to exclude confirmation bias. This was rarely the case in peptide internalization studies, partly because the specificity of some commercially available inhibitors is questionable, and their targets are multifunctional. For instance, formin-dependent actin remodeling contributes to not only phagocytosis, but also endocytosis [237] and probably TNT formation, which raises concerns about the usability of SMIFH2 for phagocytosis verification [227,228]. The actin polymerization inhibitor cytochalasin B seems suitable for preliminary verification of TNT-based transport [121]. However, this inhibitor also affects other actin-dependent pathways, including endocytosis and phagocytosis, though to a lesser extent than its more potent analog cytochalasin D. Ongoing studies reveal new aspects of cytochalasin B interference with the actin network [238], suggesting the possibility of its fine-tuning or repurposing. Similarly, all pathways dependent on membrane fluidity, raft formation, and/or receptor clustering are theoretically sensitive to the cholesterol-depleting drug MβCD [165]. Thus, analyzing MβCD effects on internalization efficiency is insufficient for conclusively discriminating pathways. Finally, dynasore, a potent inhibitor of dynamin polymerization, has also been shown to decrease the cholesterol fraction in the plasma membrane [239], suggesting potential interference with raft formation along with CvME.

Additional criteria could also be considered, such as sensitivity to temperature alterations within the physiological range, which are typical of phagocytosis and endocytosis [240]. Thus far, these studies have only conducted on α-syn fibrils to confirm CME [146]. Another general problem is the focus on the early entry stages, such as membrane reshaping. This is probably because the early stages can be modeled in silico and in vitro using artificial objects, such as liposomes or vehicles [55,56]. Verifying critical late stages, such as peptide-cargo trafficking to lysosomes, is more challenging and typically requires imaging of both the peptide and cellular organelles for colocalization analysis. In this regard, the rational choice of the peptide labeling scheme is important because it can alter the internalization pathway, as has been demonstrated for various fluorescent derivatives and conjugates of oligo-R [241].

**Table 2:** Examples of (poly)peptide-based vehicles and their established intracellular transport pathways.

Pathway	CPPs	Coacervates	Fibrils	Inhibitor (Inhibited Pathway Step)
Direct and TNT-dependent transport	Penetratin [97,112]; TAT [97,102]; oligo-R (R9) [97,101,102,107]; MPG [22]; Pep-1 [22]; TP10 [111]; mR8 [101]	sELP-ON [185] and probably others [215] *	α-syn [115,118]; Tau [114]; β-amyloid [114]	Cytochalasin B (actin polymerization for TNT formation) [146,147]
Clathrin- and dynamin-mediated endocytosis	Oligo-R (R8) [242]; TAT [122]; TP10 [243] and its phosphorylated derivatives [135]; P-rich (XZZ)_3_ [23]	-	α-syn [119]; Tau [142]; β-amyloid [142]; Huntingtin [143]	Chlorpromazine (clathrin assembly [132]) and dynasore (GTP-dependent dynamin polymerization [133])
Caveolin- and raft-mediated endocytosis	TAT [172,244]; TP10 [174]	HB*pep* [182,183]; (GHGLY)_4_ and (GHGVY)_4_ [60]	α-syn [144,150,245]; PrP^Sc^ [246]; Tau [149]; β-amyloid [149]	MβCD (cholesterol-driven raft formation [165])
Macropinocytosis	TAT [122,207]; oligo-R (R8) [122,208,209]; oligo-R (R12) [203]; PF14 [24]	HB*pep* [214]; (RRASL)_1–3_ [184]; sELP-ON [185]	α-syn [217]; Tau [142,216,217]; β-amyloid [217]; Huntingtin [143]	Amiloride and EIPA (pH control by Na^+^/H^+^ exchangers that enables membrane remodeling [212]) and wortmannin (PI3K activity that enables diacylglycerol- and Ras-dependent membrane remodeling [213])
Phagocytosis	-	HB*pep* [181]	β-amyloid [232]; α-syn [230]	SMIFH2 (formin-dependent actin polymerization [227,228]) and CK666 (Arp2/3-dependent nucleation of actin polymerization [229])

* Direct transport is presumably facilitated by extensive membrane ruffling and accompanies the macropinocytosis-like uptake.

## 9. Conclusions

CPPs have proven to be integral to the intracellular delivery of therapeutic agents and molecular tools. The mechanisms of their direct transport, in the absence of cargo molecules, are relatively well understood, at least for cationic and amphipathic CPPs. However, the addition of cargo allows for endocytosis and raises concerns about cargo trapping or digestion in endosomes or lysosomes. New types of (poly)peptide-based vehicles, such as coacervates and fibrils, exhibit unique internalization patterns that seem to depend on the physical state and structural features of the vehicle rather than the cargo. Fibrils appear to exploit all canonical pathways, at least to some extent, except for direct transport via membrane pore stabilization or inverted micelle formation, which is characteristic of monomeric/oligomeric CPPs. Coacervates exhibit unique, poorly characterized entry pathways resembling raft-dependent endocytosis, micropinocytosis, and/or phagocytosis. Categorizing the entry pathways largely relies on uptake sensitivity to a limited number of presumably specific inhibitors, which may cause artifacts in the case of mixed or unique pathways. Nevertheless, multiple studies support the lack of lysosomal trapping of coacervate-delivered cargo, at least for classical pentapeptide-based coacervates. Together with tunable design and the lack of immunogenicity, this makes coacervates an exciting alternative to classical drug carriers. In contrast, fibrils are immunogenic and show promise as antigen carriers for vaccine development. However, further elucidation of their intracellular entry and distribution is needed, which would probably benefit from expanding the panel of specific inhibitors to cover several steps of the complex internalization pathways.

## Figures and Tables

**Table 1:** Characteristic features of CPPs, coacervates and fibrils.

	Phase State		
	CPPs	Coacervates	Fibrils
Size	<5 nm	300 nm–1 µm	length: up to several μm width: 2–7 nm (protofilaments); 7–20 nm (filaments)
Charge and/or sequence patterns	Positive or weakly positive charge	Charge blockiness; repeated sequences	The presence of amyloidogenic patterns (alternating blocks of polar and hydrophobic residues)
Hydrophobicity	Amphipathic or hydrophobic	Hydrophobic or amphipathic	Amphipathic or hydrophobic
Secondary structure	Disordered or α-helical	Disordered	Cross-β structure
Immunogenicity	Non-immunogenic	Non-immunogenic	Can be immunogenic
Toxicity	Non-toxic	Non-toxic	Can be toxic
The most common entry pathway without cargo	Direct transport	Cholesterol-dependent endocytosis/ phagocytosis-like mechanism	HSPG-dependent endocytosis

## Data Availability

No new data were created or analyzed in this study. Data sharing is not applicable to this article.

## Abbreviations

The following abbreviations are used in this manuscript:

CPP	Cell-penetrating peptide
TAT	HIV transactivator of transcription
pANTP	Penetratin
LLPS	liquid–liquid phase separation
TNT	Tunneling nanotube
CME	Clathrin-mediated endocytosis
CvME	Caveolin-mediated endocytosis
HSPG	Heparan sulfate proteoglycan
MβCD	Methyl-β-cyclodextrin
GPI	Glycosylphosphatidylinositol
EHD2	EH domain-containing protein 2
LAG3	Lymphocyte-activation gene 3
TLR	Toll-like receptor
Cdc42	Cell division control protein 42
EIPA	Ethylisopropylamiloride
PI3K	Phosphatidylinositol 3-kinases
